# Host–Guest Complexation of Olmesartan Medoxomil by Heptakis(2,6-di-O-methyl)-β-cyclodextrin: Compatibility Study with Excipients

**DOI:** 10.3390/pharmaceutics16121557

**Published:** 2024-12-04

**Authors:** Dana Emilia Man, Ema-Teodora Nițu, Claudia Temereancă, Laura Sbârcea, Adriana Ledeți, Denisa Ivan, Amalia Ridichie, Minodora Andor, Alex-Robert Jîjie, Paul Barvinschi, Gerlinde Rusu, Renata-Maria Văruţ, Ionuț Ledeți

**Affiliations:** 1 Faculty of Medicine, “Victor Babeş” University of Medicine and Pharmacy, 2 Eftimie Murgu Square, 300041 Timisoara, Romania; man.dana@umft.ro (D.E.M.); andor.minodora@umft.ro (M.A.); 2 Faculty of Pharmacy, “Victor Babeş” University of Medicine and Pharmacy, 2 Eftimie Murgu Square, 300041 Timisoara, Romania; ema-teodora.nitu@umft.ro (E.-T.N.); afulias@umft.ro (A.L.); circioban.denisa@umft.ro (D.I.); amalia.ridichie@umft.ro (A.R.); alex-robert.jijie@umft.ro (A.-R.J.); ionut.ledeti@umft.ro (I.L.); 3 Advanced Instrumental Screening Center, Faculty of Pharmacy, “Victor Babeș” University of Medicine and Pharmacy, 2 Eftimie Murgu Square, 300041 Timisoara, Romania; 4 Faculty of Industrial Chemistry and Environmental Engineering, University Politehnica Timisoara, 2 Victoriei Square, 300006 Timisoara, Romania; claudia.temereanca@student.upt.ro (C.T.); gerlinde.rusu@upt.ro (G.R.); 5 Faculty of Physics, West University of Timisoara, 4 Vasile Parvan Blvd, 300223 Timisoara, Romania; pc_barvi@yahoo.fr; 6 Faculty of Pharmacy, University of Medicine and Pharmacy Craiova, 2-4 Petru Rares Str., 200349 Craiova, Romania; renata.varut@umfcv.ro

**Keywords:** olmesartan medoxomil, excipients, cyclodextrins, inclusion complex, solubility improvement, compatibility, thermal methods, spectroscopic techniques

## Abstract

**Background:** Olmesartan medoxomil (OLM) is the prodrug of olmesartan, an angiotensin II type 1 receptor blocker that has antihypertensive and antioxidant activities and renal protective properties. It exhibits low water solubility, which leads to poor bioavailability and limits its clinical potential. To improve the solubility of OLM, a host–guest inclusion complex (IC) between heptakis(2,6-di-O-methyl)-β-cyclodextrin (DMβCD) and the drug substance was obtained. Along with active substances, excipients play a crucial role in the quality, safety, and efficacy of pharmaceutical formulations. Therefore, the compatibility of OLM/DMβCD IC with several pharmaceutical excipients was evaluated. **Methods:** IC was characterized in both solid and liquid states, employing thermoanalytical techniques, universal-attenuated total reflectance Fourier-transform infrared spectroscopy, powder X-ray diffractometry, UV spectroscopy, and saturation solubility studies. Compatibility studies were carried out using thermal and spectroscopic methods to assess potential physical and chemical interactions. **Results:** The 1:1 OLM:DMβCD stoichiometry ratio and the value of the apparent stability constant were determined by means of the phase solubility method that revealed an *A_L_*-type diagram. The binary system showed different physicochemical characteristics from those of the parent entities, supporting IC formation. The geometry of the IC was thoroughly investigated using molecular modeling. Compatibility studies revealed a lack of interaction between the IC and all studied excipients at ambient conditions and the thermally induced incompatibility of IC with magnesium stearate and α-lactose monohydrate. **Conclusions:** The results of this study emphasize that OLM/DMβCD IC stands out as a valuable candidate for future research in the development of new pharmaceutical formulations, in which precautions should be considered in choosing magnesium stearate and α-lactose monohydrate as excipients if the manufacture stage requires temperatures above 100 °C.

## 1. Introduction

**Figure 1:**
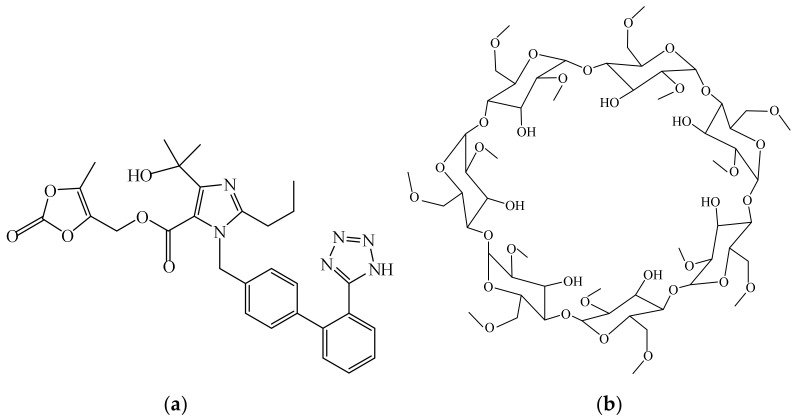
Chemical structures of OLM (**a**) and DMβCD (**b**).

Olmesartan medoxomil (OLM), *(5-methyl-2-oxo-2H-1,3-dioxol-4-yl)methyl 4-(2-hydroxypropan-2-yl)-2-propyl-1-{[2′-(1H-1,2,3,4-tetrazol-5-yl)-[1,1′-biphenyl]-4-yl]methyl}-1H-imidazole-5-carboxylate* (Figure 1a), is an imidazole derivative belonging to the group of angiotensin II receptor blockers used in hypertension [1,2]. OLM also exerts renal protective effects, antioxidant activity [3], and anti-atherosclerotic effects [4] and improves endothelial function [5], and recent studies highlight its potential for repurposing in medical conditions such as ulcerative colitis, cancer, and Alzheimer’s disease [2,6,7,8]. It is an ester-type prodrug that undergoes hydrolysis during the absorption process from the gastrointestinal tract to form the active metabolite, olmesartan [9,10]. OLM belongs to BCS (Biopharmaceutics Classification System) class II, exhibiting a very low aqueous solubility (7.75 mg L^−1^ at 25 °C) and high lipophilicity (log *P* of 4.31). After oral administration, it suffers an extensive first-pass effect, with its bioavailability being approximately 26% [9,10,11].

Cyclodextrins (CDs) are water-soluble cyclic oligosaccharides built up from D-glucopyranose units connected by α-(1,4)-glycosidic bonds; they are a valuable subject of many studies due to their broad applications in biomedicine; in pharmaceutical, food, textile, and cosmetic fields; and in analytical chemistry and agriculture production. CDs present a particular truncated-cone-shape structure with a hydrophilic shell responsible for their water solubility and a hydrophobic core that enables them to fully or partially encapsulate hydrophobic drug substances with appropriate size, giving rise to inclusion complex (IC) formation [12,13,14,15,16]. Natural CDs are made of six, seven, and eight glucose units referred to as α, β, and γ, and they are considered as GRAS (Generally Regarded/Recognized as Safe) by the FDA (Food and Drug Administration) and also approved for use by the EMA (European Medicines Agency) [17,18,19]. The host–guest IC formation remarkably improves the physicochemical properties and biopharmaceutical profile of the guest molecule; in pharmaceutical applications, the main purpose of CD use derives from their solubilizing effect on the poorly water-soluble drug substances, especially those belonging to BCS classes II and IV [19,20,21,22,23,24,25]. CDs provide additional benefits such as improving biological membrane permeability, altering the drug-release profile, enhancing the physical and chemical stability of the embedded compounds and their biological and antioxidant activity, masking unpleasant smells and tastes, slowing down irritation at the administration site, and preventing drug–excipient or drug–drug interaction [26,27,28,29,30,31,32,33]. Among these pharmaceutical excipients, β-CD is one of the most frequently used due to its availability and internal cavity size appropriate for a large range of drug substances. However, it exhibits low aqueous solubility and, to overcome this limitation, CD derivatives have been obtained, such as heptakis(2,6-di-O-methyl)-β-cyclodextrin (DMβCD) (Figure 1b) that possesses improved water solubility and inclusion abilities [34,35]. DMβCD is also used as a culture medium modifier in the manufacturing of pertussis antigens for promoting the growth of bacteria [36].

Different strategies have been adopted in order to improve OLM physicochemical and biopharmaceutical profiles, such as the development of OLM-loaded lipid-based nanoformulations [1]; extrudes containing OLM in amorphous form obtained using the hot-melt extrusion technique [37]; extended-release tablets using chitosan/sulfobutyl ether-β-cyclodextrin composites [38]; the preparation of OLM-lipid-based formulations with Acrysol EL135, Tween 80, and Transcutol P [10]; OLM ternary solid dispersions containing hydroxypropyl-β-cyclodextrin and N-methyl-D-glucamine [9]; and OLM complexation with CDs [38,39,40]. Thakkar et al. reported the inclusion of OLM into the hydroxypropyl-β-cyclodextrin cavity [39], and in our previous paper, we investigated OLM encapsulation in two methylated β-CDs, randomly methylated β-cyclodextrin (RM-β-CD) and heptakis(2,3,6-tri-O-methyl)-β-cyclodextrin (TM-β-CD) in both solution and solid states [40].

Pharmaceutical dosage forms are the result of the combination of active pharmaceutical ingredients (APIs) and excipients, which facilitate the manufacture, stability, patient compliance, and administration of drugs. An appropriate pharmaceutical formulation highly depends on the rigorous selection of excipients; even if these auxiliary substances are considered inert molecules, they can physically or chemically interact with APIs during manufacturing, packaging, and storage processes, affecting the bioavailability, stability, potency, and safety of drug products [41,42,43,44]. Thus, the quality, efficiency, stability, and safety of dosage forms are strongly dependent on the assessment of potential interaction between API and excipients. According to International Conference on Harmonisation (ICH) Q8 recommendations, the compatibility between drug substances and excipients should be investigated as a part of pharmaceutical development [45,46].

In this framework and taking into account the clinical potential of OLM molecule owing to its multifaceted effects, it is of particular relevance to obtain new pharmaceutical formulations of this drug substance with improved bioavailability. In the present study, we aimed to assess the inclusion complexation of OLM by DMβCD to enhance drug solubility and to investigate the compatibility of the obtained supramolecular entity with pharmaceutical excipients commonly used in pharmaceutical formulations, namely talc (TA), starch (STA), *α*-lactose monohydrate (LA), and magnesium stearate (MgSTR). According to our knowledge, the compatibility of OLM/CD ICs with excipients has not been evaluated so far. The OLM/DMβCD IC was characterized in liquid and solid states by means of phase solubility analysis, UV spectroscopy, universal-attenuated total reflectance–Fourier-transform infrared (UATR-FTIR) spectroscopy, powder X-ray diffractometry (PXRD), thermogravimetry (TG), derivative thermogravimetry (DTG), and differential scanning calorimetry (DSC). Saturation solubility studies were also performed to evaluate the solubility profile of the supramolecular adduct. Theoretical studies were carried out employing molecular modeling in order to investigate the geometry of the IC. The compatibility of OLM/DMβCD IC with excipients was investigated by means of thermal and spectroscopic methods.

## 2. Materials and Methods

### 2.1. Materials

Olmesartan medoxomil (as the Pharmaceutical Secondary Standard) (PHR1851) was acquired from Sigma-Aldrich and heptakis(2,6-di-O-methyl)-β-cyclodextrin (CYL-4668) was acquired from Cyclolab R&L Ltd. (Budapest, Hungary), which were used as received. The excipients (pharmaceutical grade) used in this study were talc (#BCBQ7164V) from Sigma (Germany), starch (SZBF167) from Grain Processing Corporation (USA), magnesium stearate (#SZBF2590V) from Sigma (Germany), and *α*-lactose monohydrate (#SLBK4809V) from Sigma (Germany). All other chemicals and reagents used were of analytical purity.

### 2.2. Preparation of the Solid Inclusion Complex and Physical Mixtures with Excipients

To obtain the OLM/DMβCD inclusion complex, the kneading method was employed with a guest/host molar ratio of 1:1. To this end, amounts of 0.3549 g of OLM and 0.8456 g of DMβCD were pulverized using an agate mortar and triturated with a mixture of ethanol/distilled water (1:1, m/m) to obtain a paste that was kneaded for 45 min, adding an appropriate amount of the solvent system during this process to maintain its suitable consistency. After that, the final product was dried at room temperature and subsequently in an oven at 40 °C for 24 h. The kneaded product (KP) thus obtained was pulverized and passed through a 75 µm size sieve. In addition, a physical mixture (PM) of OLM and DMβCD was obtained in the same molar ratio as KP by mixing the substances in an agate mortar with a pestle for 10 min with no solvent added. Then, PM was passed through a 75 µm size sieve.

Subsequently, the physical mixtures of OLM/DMβCD IC with each tested excipient were obtained in the ratio of 1:1 (m/m) by mixing in an agate mortar for 5 min.

### 2.3. Phase Solubility Studies

The method reported by Higuchi and Connors [47] was employed to perform the phase solubility analysis. To this end, an excess amount of OLM was added to 3 mL of a series of DMβCD solutions with concentrations ranging from 0 to 25 mM in 0.1 M phosphate buffer, pH 7.4. The obtained suspensions were shaken for 5 days at 25 °C to reach equilibrium and then were filtered using a 0.45 µm nylon disk filter. The solutions were appropriately diluted and the OLM concentration was assessed by means of UV spectrophotometric measurements at 257 nm. A Jena Analytik Specord 250 Plus double-beam spectrophotometer (Jena, Germany) with matched quartz cells of 1 cm was employed for recording the UV spectra.

The apparent stability constant (*K*_1:1_) of the OLM/DMβCD system was determined from the phase solubility diagram using the following equation:

(1) $$K_{1:1}=\frac{Slope}{S_{0}\;\left(1-Slope\right)}$$

where *S*_0_ is the solubility of OLM in 0.1 M phosphate buffer, pH 7.4, without DMβCD.

### 2.4. Encapsulation Efficiency and Loading Efficiency Analysis

An amount of 0.0103 g of OLM/DMβCD IC was accurately weighed and mixed with 5 mL of phosphate buffer 0.1 M, pH 7.4, in a 10 mL volumetric flask, and then the solvent was added up to the mark. After the sonication of the obtained solution for 10 min, it was filtered using a 0.45 µm nylon disk filter and suitably diluted for the quantification of OLM by means of UV spectroscopy.

The Encapsulation Efficiency (EE) and Loading Efficiency (LE) were estimated using Equations (2) and (3) [33,48]:

(2) $$EE\%=\frac{M_{E}}{M_{T}}\cdot 100$$

(3) $$LE\%=\frac{M_{E}}{M_{I}}\cdot 100$$

where *M_E_* is the amount of encapsulated OLM, *M_T_* is the total amount of OLM added initially during the obtaining of IC, and *M_I_* is the mass of IC.

### 2.5. Molecular Modeling Studies

To explore the interaction between OLM and DMβCD, molecular docking techniques were applied. The three-dimensional coordinates of OLM were obtained from PubChem (CID 130881), and its geometry was optimized using density functional theory (DFT) with the B3LYP functional and 6–311 G basis set. The DMβCD structure was constructed based on the curated coordinates of the ligand 2QKH (determined by X-ray diffraction at a resolution of 1.9 Å), sourced from the Protein Data Bank database [49]. Methyl groups were manually added to the free hydroxyl groups at the 2 and 6 positions of β-CD using GaussView 5 (Semichem Inc., Shawnee Mission, KS, USA), and the resulting structure was similarly optimized with the DFT/B3LYP/6-311G method.

Phase solubility studies revealed a 1:1 molar ratio for the OLM:DMβCD IC stoichiometry. During the docking simulations, OLM was designated as the ligand, while DMβCD served as the receptor to identify the optimal IC configuration and compute the total affinity energy (kcal mol^−1^). Docking simulations were conducted using Autodock 4.2.6 software with AutoDockTools [50], and all calculations were performed under vacuum conditions. The Lamarckian genetic algorithm was employed, with a population size of 150 and 30 simulation runs, while default settings were retained for other parameters. Visual representations of molecular models were generated using PyMol (http://www.pymol.org, accessed on 25 June 2024) [51].

### 2.6. Thermal Investigations

A Setline TGA instrument (SETARAM, Caluire, France) was used to investigate the parent compounds, OLM and DMβCD, OLM/DMβCD KP, PM, and the mixtures of KP with each excipient. Samples (around 3–4 mg) in alumina crucibles were heated in an air atmosphere with a heating rate of 10 °C min^−1^ at a flow rate of 100 mL min^−1^ from 30 to 450 °C.

### 2.7. FTIR Spectroscopy

A Shimadzu IRTracer-100 FT-IR spectrometer with an ATR device was used to perform UATR-FTIR spectroscopic investigations. The spectral data were obtained directly from solid samples in the spectral range of 4000–400 cm^−1^. Spectra were built up after a 16 co-added acquisitions, with a spectral resolution of 2 cm^−1^.

### 2.8. Powder X-Ray Diffraction Analysis

A Bruker D8 Advance powder X-ray diffractometer was used to record the PXRD pattern of OLM, DMβCD, their KP and PM, and the mixtures of KP with selected excipients. The PXRD spectra of the samples were recorded at ambient temperature between 10 and 45° (2*θ*), using CuKα radiation (40 kV, 40 mA) and a Ni filter.

### 2.9. Solubility Profile Evaluation for OLM/DMβCD Kneaded Product

The solubility profile of OLM upon the complexation process was assessed by the saturation shake-flask technique. To this end, saturated solutions of OLM, OLM/DMβCD PM, and KP were obtained by adding an excessive amount of compounds to 5 mL of 0.1 M phosphate buffer, pH 7.4. These solutions were stirred for 24 h at room temperature and, after that, were filtered (0.45 µm nylon disk filter). The filtrates were suitably diluted and their absorbance was recorded at 257 nm. OLM quantification was carried out using the calibration curve, as in our previous study [40].

## 3. Results and Discussion

### 3.1. Phase Solubility Studies

**Figure 2:**
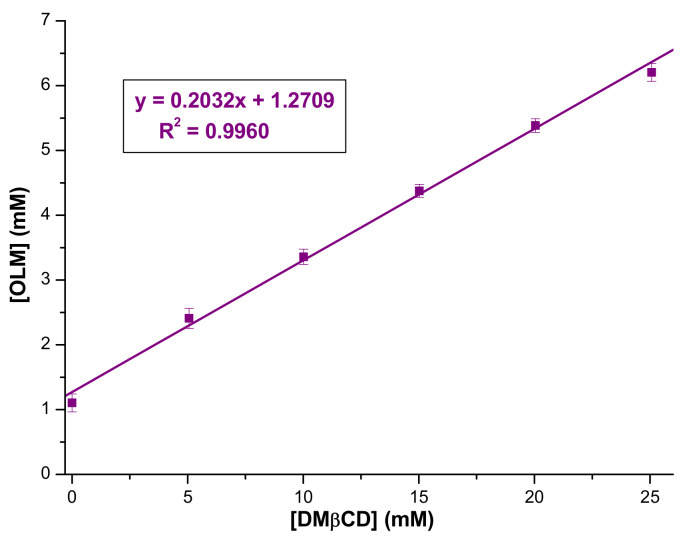
Phase solubility diagram of OLM with DMβCD in 0.1 M phosphate buffer, pH 7.4.

Phase solubility studies were carried out to investigate the molar ratio of the parent compounds in the IC and to determine the value of the apparent stability constant. Figure 2 depicts the phase solubility diagram of OLM at different DMβCD concentrations in 0.1 M phosphate buffer (pH 7.4) at 25 °C. As Figure 2 reveals, the OLM apparent solubility increases linearly (R^2^ = 0.9960) as a function of DMβCD concentration in the 0–25 mM concentration range, which can be attributed to an increasing amount of drug substance embedded in the CD cavity as the DMβCD concentration increases, thus demonstrating the interaction between OLM and DMβCD. The linear relationship between the host concentration and the guest solubility indicates an *A_L_*-type diagram according to the Higuchi and Connors classification [47]. This diagram along with its slope value less than unity (0.2032) suggests the formation of a soluble IC between OLM and DMβCD in a 1:1 molar ratio. The stability constant (*K*_1:1_) of the OLM/DMβCD IC obtained using the data from the phase solubility diagram and Equation (1) is 231.10 ± 2.97 M^−1^. This parameter is of great importance, providing insights into the binding strength between the guest and the host; a value ranging between 100 and 5000 M^−1^ is considered ideal for the formation of an IC that may enhance the bioavailability [52,53]. Therefore, the obtained value confirms the stability and applicability of the IC in increasing OLM solubility and, accordingly, its bioavailability. Comparing this value with the ones obtained for the IC of OLM with RM-β-CD and TM-β-CD [40], it can be noticed that the interaction between the drug substance and CDs, and, consequently, the IC stability, decrease as follows: DMβCD > RM-β-CD > TM-β-CD.

### 3.2. Encapsulation Efficiency and Loading Efficiency Analysis

The OLM content in the IC was assessed using UV-spectrophotometric measurement at 257 nm. The values of EE and LE for OLM/DMβCD were determined to be 86.52 ± 2.54% and 25.45 ± 1.16%, greater than those found in our previous study [40], when RM-β-CD and TM-β-CD were used as host molecules. These data are in agreement with the results of phase solubility analysis, where the greatest value for the apparent stability constant was also found for DMβCD when compared with RM-β-CD and TM-β-CD, revealing a better interaction between OLM and DMβCD. This phenomenon might be attributed to the changes in flexibility of C and O atoms from the CDs by methylation [54], with the greatest increase in flexibility for TM-β-CD. Moreover, the presence of two adjacent methyl groups at O_2_ and O_3_ results in some repulsion, thus preventing the decrease in area of the secondary hydroxyl rim (associated with methylation process), and lowering the stability constant of the IC [55]. All these lead to less effective retention of the guest molecule and weaker interaction between the guest and the host molecules.

### 3.3. Molecular Modeling Studies

Molecular docking was employed to determine the potential conformation of the IC; it predicted non-covalent interactions between guest and host molecules in the IC obtained in a molar ratio of 1:1. The molecular modeling studies were carried out using Autodock 4.2.6 software together with AutoDockTools [50].

AutoDock is a suite of automated docking tools aimed to predict the binding manner of small molecules, such as substrates or drug candidates to a receptor of known 3D structure. According to AutoDock, the binding energy represents the sum of the intermolecular forces acting on the receptor–ligand complex.

Binding energy = A + B + C − D

where A stands for the final intermolecular energy + van der Waals energy + hydrogen bonds + desolvation energy + electrostatic energy (kcal mol^−1^), B represents the final total internal energy (kcal mol^−1^), C is the torsional free energy (kcal mol^−1^), and D is the energy of the unbound system (kcal mol^−1^).

The binding free energy for OLM/DMβCD IC was calculated to be −6.74 kcal mol^−1^. Root-mean-square deviation (RMSD) values were calculated after Autodock 4.2.6 redocking, and the obtained low values (all of them ≤ 0.25 Å) suggest the robustness of our docking methodology.

**Figure 3:**
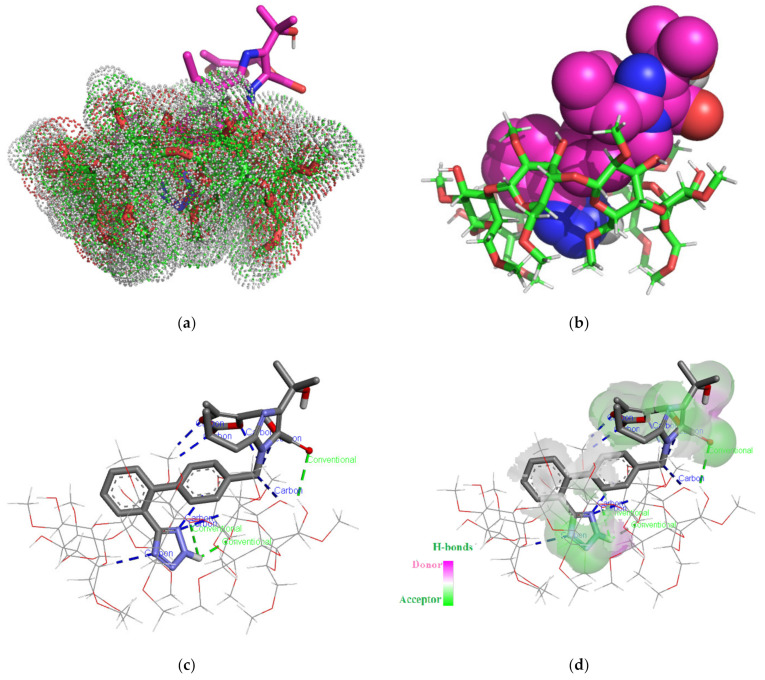
OLM/DMβCD IC simulation for 1:1 molar ratio. Images (**a**,**b**) show the supramolecular entity from the secondary face of the DMβCD cavity. OLM is represented as sticks colored by element, and DMβCD is represented by red/green/white dots (**a**); OLM is shown as spheres colored by element, and DMβCD is shown as sticks in red/green/white (**b**). Polar/hydrophobic contacts between OLM and DMβCD, where OLM is represented as sticks colored by element, and DMβCD is represented as lines (**c**). H-bond surface interaction of OLM/DMβCD (**d**).

The theoretical OLM/DMβCD chemical entity as rendered in the PyMOL molecular visualization system [51] simulated in a molar ratio of 1:1 is shown in Figure 3.

The analysis of the 3D images of OLM/DMβCD (1:1) interaction unveiled the formation of three classical hydrogen bonds that implies the 2H hydrogen of the tetrazole heterocycle, carbonyl oxygen of the carboxylate radical from the OLM structure, and the oxygen/hydrogens of DMβCD glucopyranose units with lengths of 2.1 Å, 2.77 Å, and 3.09 Å. Eight intermolecular non-classical hydrogen bonds with the length varying between 2.28 Å and 3.29 Å were formed, involving tetrazole and 2-oxo-1,3-dioxol OLM heterocycles and the hydrogen atoms from the pyranose ring of carbohydrate moieties.

### 3.4. Characterization

#### 3.4.1. Thermal Analysis

**Figure 4:**
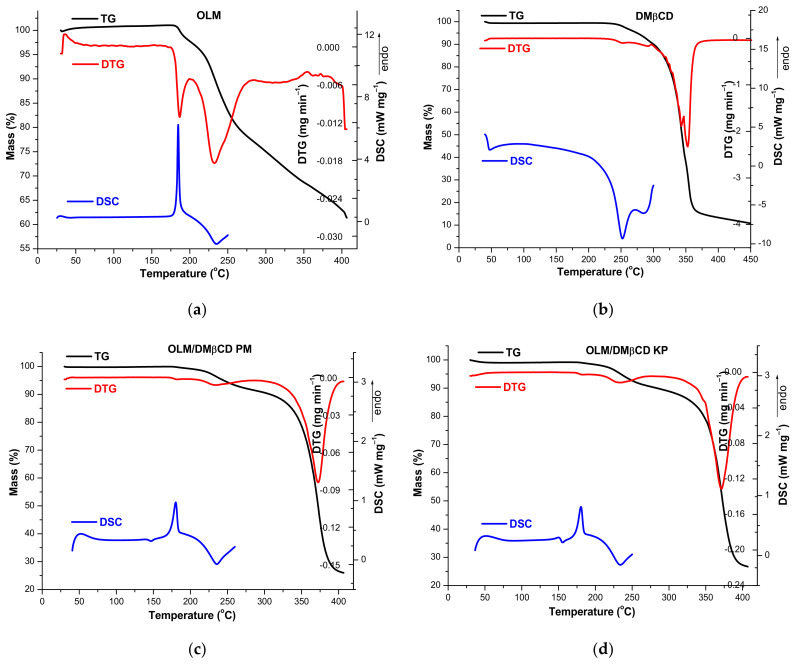
TG/DTG/DSC thermoanalytical curves of OLM (**a**); DMβCD (**b**); OLM/DMβCD PM (**c**); and KP (**d**) in air atmosphere.

The thermal behavior of parent compounds and their binary products PM and KP, respectively, was investigated using TG, DTG, and DSC, and the thermoanalytical curves of OLM, DMβCD, OLM/DMβCD KP, and PM binary systems are depicted in Figure 4.

The TG curve of OLM reveals a very small mass increase process between 30 and 172 ºC (Δ*m* = 1.0%) (Figure 4a) due to the oxidative degradation of the drug substance that most probably results in the cleavage of the 2-oxo 1,3-dioxol heterocycle. Starting with 172 °C, a continuous mass loss is noticed up to 400 °C in the TG curve of OLM (Δ*m* = 38.5%) as a result of thermally induced degradation that takes place in four stages according to the DTG curve. The first stage appears in the temperature range of 172–202 °C (DTG_peak_ = 187 °C, Δ*m* = 3.5%), the second is noticed between 202 and 283 °C (DTG_peak_ = 232 °C, Δ*m* = 20.3%), and the third process is observed from 283 °C to 355 °C (DTG_peak_ = 322 °C, Δ*m* = 9.0%). Above 355 °C, the mass loss process continues until a residual mass of 61.5% at 400 °C. The DSC curve of OLM shows a sharp endothermic peak with a maximum at 185 °C corresponding to the melting of the drug substance followed by an exothermic one (DSC_peak_ at 234 °C) attributed to the OLM decomposition.

DMβCD presents a considerable thermal stability since its decomposition starts around 226 ºC as revealed by its thermal profile (Figure 4b). The DTG curve shows two peaks corresponding to the two steps of decomposition attributed to the thermo-oxidation processes. The first one is observed in the temperature range of 226–347 °C (DTG_peak_ = 343 °C Δ*m* = 57.0%) and the second one is noticed between 347 and 382 °C (DTG_peak_ = 353 °C Δ*m* = 28.5%). Above 382 ºC, the mass loss continues until a residual mass of 13.4% at 400 °C. The thermo-oxidative degradation of DMβCD is also highlighted on the DSC curve by the exothermic events from 252 and 286 °C.

The thermal profiles of the binary systems show significant differences from those of the parent substances. A stability stage is noticed in TG curves of both PM (Figure 4c) and KP (Figure 4d) up to 120 °C, and then a very small mass increase is noticed between 120 and 155 °C for KP (Δ*m* = 0.10%) and between 120 and 171 °C for PM (Δ*m* = 0.14%). This mass increase is probably the result of the thermally induced oxidation of the 2-oxo 1,3-dioxol heterocycle, which are not entrapped in the DMβCD cavity, as the molecular modeling study revealed. Above these temperature values, decomposition accompanied by mass loss are observed in both binary systems in two steps, as the DTG curves point out. The first one is noticed in the temperature ranges of 165–291 °C for KP (DTG_peak_ = 234 °C Δ*m* = 9.7%) and 171–291 °C for PM (DTG_peak_ = 233 °C Δ*m* = 8.8%) and the second appears between 291 and 400 °C for both KP (DTG_peak_ = 371 °C Δ*m* = 62.3%) and PM (DTG_peak_ = 372 °C Δ*m* = 64.6%). Analyzing the DTG curves of the binary systems, the disappearance of OLM characteristic DTG_peaks_ from 187 and 322 °C can be observed in both KP and PM. The residual mass at 400 °C is 27.25% in the case of KP and 26.54% for PM. The endothermic peak attributed to OLM melting shows a significant reduction in the DSC curves of the binary systems, greater in the case of KP, and is also shifted to smaller temperature values (DSC_peaks_ at 180.1 and 180.4 °C) in both PM and KP curves.

These results clearly demonstrate the existence of an interaction between OLM and DMβCD in both KP and PM, supporting the formation of the host–guest IC. OLM interactions with other CDs in the physical mixture even in the case of PM prepared without grinding, but only gentle mixing with a spatula, were reported earlier [39,40].

#### 3.4.2. FTIR Spectroscopy

**Figure 5:**
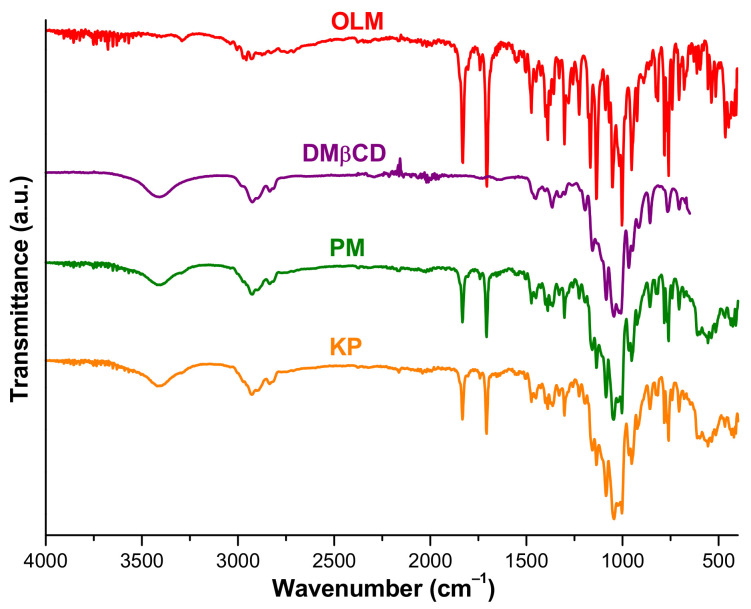
FTIR spectra of OLM, DMβCD, OLM/DMβCD PM, and KP.

FTIR spectroscopy is a powerful tool in assessing the guest–host interaction by monitoring the absorption changes of functional groups of guest and host molecules. The FTIR spectra of the investigated compounds are depicted in Figure 5.

The spectral pattern of OLM exhibits characteristic absorption bands at 1830, 1705, 1225, 1167, 1551, 1503, 1474, 1389, 1302, 1136, 1002, 953, 826, 816, 781, 770, 760, and 741 cm^−1^, which were assigned to the functional groups of drug molecule in our previous study [40].

The DMβCD FTIR spectrum shows a broad absorption band at 3408 cm^-1^ attributed to O-H stretching vibration of non-methylated hydroxyl groups and other characteristic bands at 2925 cm^−1^ assigned to the stretching vibration of C-H from CH_2_, at 2835 cm^−1^ corresponding to the stretching vibration of methyl groups, and at 1365 cm^−1^ that corresponds to C-H bending from CH_2_. Also, the absorption bands from 1156 and 1045 cm^−1^ were attributed to the C-O stretching vibration and to C-O-C stretching vibrations [31,34].

The FTIR spectra of both binary systems show significant differences when compared with those of the parent compounds. Thus, OLM characteristic absorption bands of ester and lactone C=O stretching vibrations are displaced from 1705 and 1830 cm^−1^ at 1707 and 1832 cm^−1^ in both PM and KP spectra; the OLM band corresponding to the C-O stretching of the ester group from 1167 cm^−1^ is shifted to 1157 cm^−1^ in PM and KP spectra; and the band assigned to the C=N stretching vibration from 1389 cm^−1^ is noticed at 1388 cm^−1^ in PM. Also, the OLM absorption band from 1090 is observed at 1086 cm^−1^ and the bands attributed to C_ar_-H bending from 816, 781, and 770 cm^−1^ are displaced at 818, 783, and 767 cm^−1^ in both binary product spectra. Moreover, several OLM characteristic bands are not present anymore in the FTIR patterns of both PM and KP, namely, the OLM bands from 1281, 1069, and 1016 cm^−1^. In addition, the DMβCD absorption band from 3408 cm^−1^ is shifted to 3414 cm^−1^ in PM and to 3422 cm^−1^ KP; also, the C-H stretching vibration band from CH_2_ is shifted from 2925 to 2928 cm^−1^ in the KP spectrum and the band of C-O-C stretching vibrations from 1045 cm^−1^ is displaced at 1043 cm^−1^ in the KP spectrum, suggesting the DMβCD involvement in hydrogen bonds with OLM.

The results provided from FTIR analysis highlight a shift to different wavenumbers for several of the characteristic absorption bands of OLM and DMβCD, along with their reduction in intensity in the spectral patterns of PM and KP and also the disappearance of some OLM characteristic bands in the spectra of the binary products. These differences in the spectral patterns of PM and KP in comparison with those of OLM and DMβCD demonstrate an interaction between the drug substance and cyclodextrin in both PM and KP, supporting the results of thermoanalytical methods. The findings of FTIR studies are also in agreement with the outcomes of molecular modeling analysis.

#### 3.4.3. Powder X-Ray Diffraction Analysis

**Figure 6:**
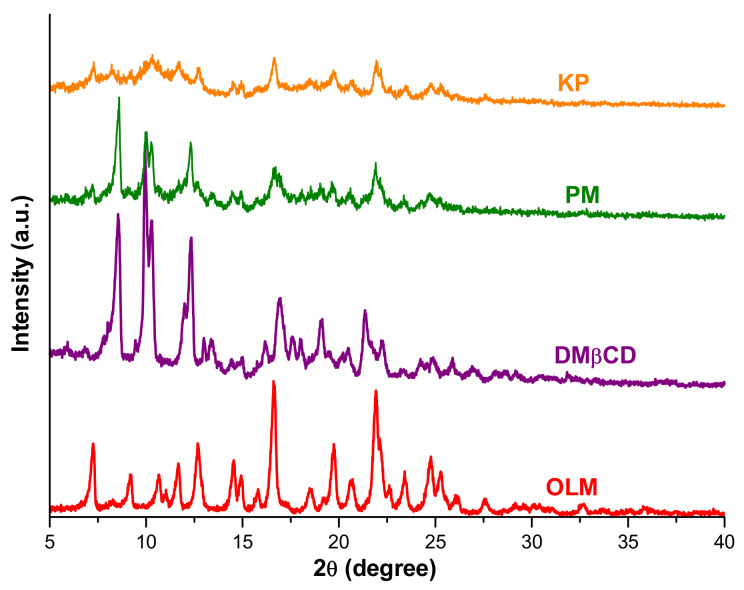
Diffraction profiles of OLM, DMβCD, and OLM/DMβCD binary systems PM and KP.

The PXRD pattern of OLM, DMβCD, OLM/DMβCD PM, and KP binary systems are presented in Figure 6.

The X-ray diffraction pattern of OLM displays two sharp characteristic reflections at 2*θ* angles of 16.61 and 21.92 and other characteristic peaks at 7.25, 9.17, 10.64, 11.69, 12.68, 14.54, 19.73, 24.77, and 25.25 2*θ* that highlight the crystalline profile of drug substance [40]. Similarly, DMβCD also exhibits sharp characteristic diffraction peaks at 2*θ* angles of 8.54, 9.95, 10.29, 12.31, 16.89, 19.04, and 21.32 due to its crystalline profile [31]. The PXRD spectrum of OLM/DMβCD PM shows a marked decrease in intensity of both OLM and DMβCD characteristic peaks together with the disappearance of OLM characteristic reflections at 9.17, 10.64, and 11.69 2*θ*. Furthermore, the OLM characteristic peak at 2*θ* angles of 14.54 is displaced at 14.42 2*θ*, and also the sharp DMβCD peaks are shifted at 8.60, 10.03, and 10.27 2*θ* in the PM diffraction pattern. The PXRD profile of OLM/DMβCD KP reveals a more prominent reduction in peak intensity for both parent compounds along with the disappearance of an important number of OLM and DMβCD characteristic peaks, highlighting the amorphization process of OLM in the KP, which in turn is expected to enhance OLM solubility. The two sharpest OLM peaks are noticed at 2*θ* angles of 16.65 and 21.94, while the sharpest DMβCD crystalline reflection together with OLM characteristic reflections at 9.17, 10.64, and 24.77 2*θ* were absent in the KP diffractometric profile.

These results provide supporting evidence for the formation of new compounds less crystalline than the parent substances alone as a result of the interaction between OLM and DMβCD in both binary systems, PM and KP, weaker in the PM system, supporting the findings of thermal analysis and FTIR spectroscopy.

#### 3.4.4. Solubility Study of the Binary Products

**Figure 7:**
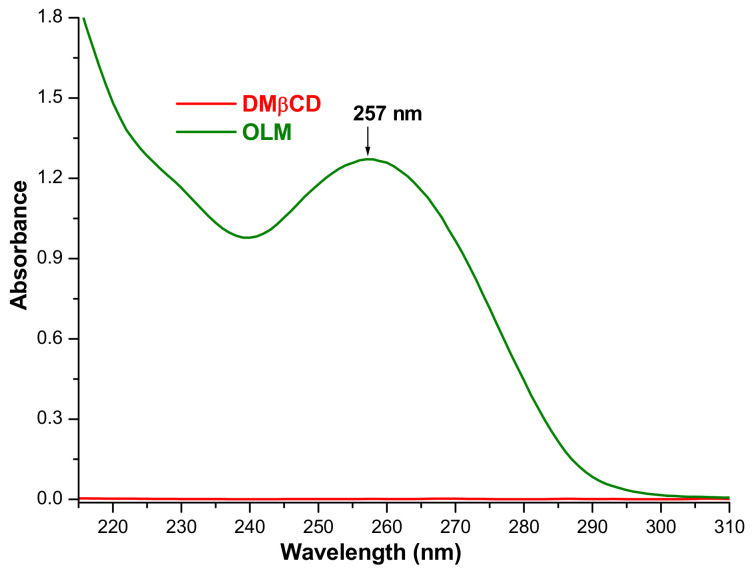
UV spectra of DMβCD 150.0 µg mL^−1^ and OLM 27.0 µg mL^−1^ in 0.1 M phosphate buffer, pH 7.4, at 25 °C.

The shake-flask method was employed to evaluate the solubility of OLM in the binary compounds [30,31,56]. As presented in our previous study, the quantification of the drug substance in saturated solutions was performed by means of UV spectroscopy and the calibration curve at 257 nm [40] since the DMβCD solution in 0.1 M phosphate buffer, pH 7.4, does not absorb UV radiation from 210 to 310 nm (Figure 7).

OLM solubility in the OLM/DMβCD supramolecular adduct obtained by the kneading method was calculated as a mean value of five determinations and was found to be 1126.30 ± 0.072 µg mL^−1^, revealing an increase in OLM solubility of 1.83-fold as compared to pure OLM (616.404 ± 0.015 µg mL^−1^). Similarly, the solubility of OLM was also assessed in the binary product PM and the calculated value was 998.25 µg mL^−1^, highlighting that the drug solubility increased by 1.60-fold in PM. These findings point out the solubilizing effect of DMβCD and also demonstrate the interaction between OLM and DMβCD in both KP and PM, sustaining the results of thermal and spectroscopic investigations.

Comparative evaluation of the OLM solubility upon complexation with DMβCD, RM-β-CD, and TM-β-CD, used in our previous study [40], highlights the decrease in the CD solubilizing effect in the following order: DMβCD > RM-β-CD > TM-β-CD. The same order for the solubilizing behavior of these methylated CDs was previously reported in the case of furosemide, tamoxifen, and amiodarone [55].

The CD solubilizing capacity on different drugs is strongly related to both their complexing ability and to the host intrinsic water solubility. The methylation of the primary hydroxyl groups results in an increase in CD binding potential, while the substitution of the secondary hydroxyl groups decreases it. Therefore, the solubilizing capacity is the consequence of these opposite effects [55].

### 3.5. Compatibility Studies with Excipients

The evaluation of potential interactions (physical and/or chemical) between the drug substance and excipients is of major importance for the development of new pharmaceutical formulations, affecting the quality, efficiency, stability, and safety of the final dosage form. Few studies are dedicated to investigating the compatibility of OLM alone or in combination with amlodipine besylate and hydrochlorothiazide with excipients [43,57], and no study assessed OLM/CD IC compatibility with excipients.

#### 3.5.1. Thermal Investigations

**Figure 8:**
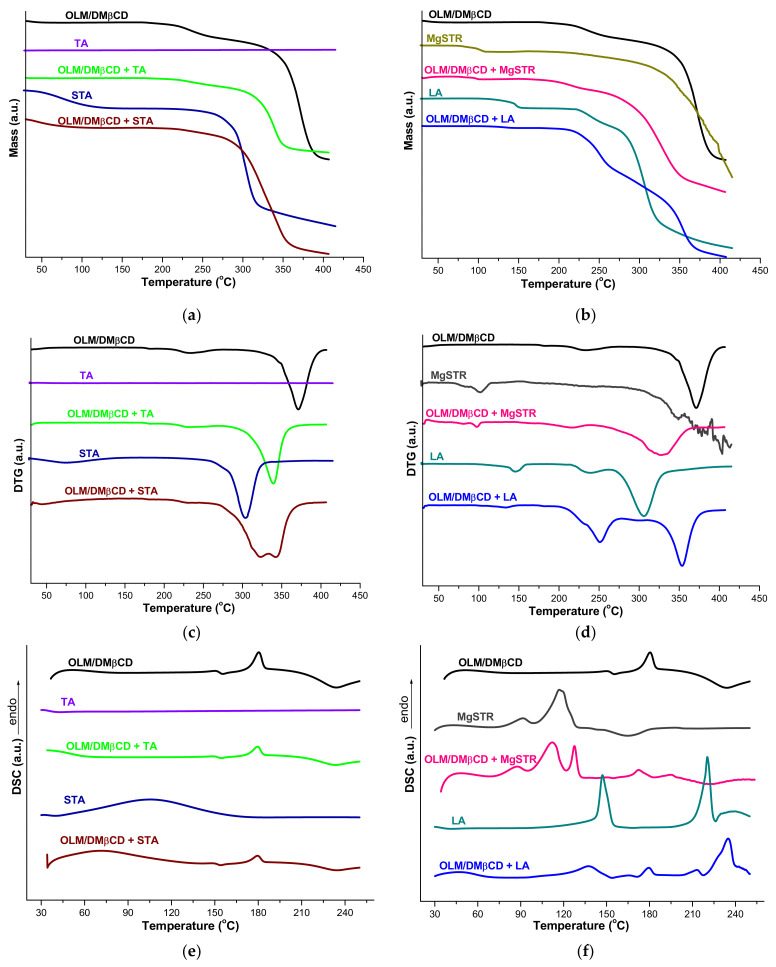
TG (**a**,**b**), DTG (**c**,**d**), and DSC (**e**,**f**) curves of OLM/DMβCD IC and its mixture with pharmaceutical excipients TA and STA (**a**,**c**,**e**), and Mg STR and LA (**b**,**d**,**f**) in synthetic air atmosphere.

The thermal behavior of OLM/DMβCD KP and its mixture with selected pharmaceutical excipients is presented in Figure 8a–f and Table 1.

According to the OLM/DMβCD KP thermoanalytical curves (TG/DTG), the thermal degradation of the supramolecular adduct is noticed in the following temperature ranges: 120–155 °C (mass increase, Δ*m* = 0.1%) followed by a narrow stability stage between 155 and 165, and then 165–291 °C (Δ*m* = 9.7%, DTG_peaks_ at 183 and 234 °C) and 291–400 °C (Δ*m* = 62.3%, DTG_peak_ at 371 °C) (Table 1). The DSC curve of the IC (Figure 8e) shows an endothermic process at 180 °C that corresponds to the melting of the drug substance, followed by an exothermic event with a maximum at 234 °C indicating KP decomposition.

The thermal profile of TA reveals the stability of the excipient in the studied temperature interval with no significant event observed on the TG/DTG/DSC curves. Regarding the mixture between OLM/DMβCD KP and TA, a stability stage is noticed up to 170 °C, when its decomposition in two steps starts, as the TG/DTG thermal curves show. The DTG_peak_ for the second decomposition step of the mixture, which corresponds to KP, is displaced to lower temperature (339 °C) in comparison with that of pure OLM/DMβCD KP (371 °C) due to the presence of TA seen as an impurity; also, a small difference between the theoretical residual mass at 400 °C (63.63%) and the experimental one (61.87%) is noticed. The OLM melting is revealed on the DSC curve of the mixture by the endothermic peak with a maximum at 180 ºC, and also, the KP decomposition is indicated by the exothermic peak (DSC_peak_ at 234 °C) (Table 1). These results indicate the lack of interactions between the IC and TA.

The TG and DTG curves of STA reveal a mass loss process between 35 and 141 °C (Δ*m* = 10.19%, DTG_peak_ at 73 °C) corresponding to the dehydration of the excipient [42] followed by decomposition in the 228–400 °C temperature range (Δ*m* = 65.64%, DTG_peak_ at 304 °C), with the residual mass at 400 °C being 24.17%. In the thermoanalytical curves of the OLM/DMβCD KP + STA, the dehydration of the excipient is followed by a stability stage in the 128–175 °C temperature range; above this temperature value, the degradations of both IC and the excipient overlap. The DTG_peak_ associated with the dehydration process of STA is shifted from 73 °C (pure excipient) to 44 °C in the DTG thermogram of the mixture, and the DTG_peaks_ for the final decomposition stage of both individual substances (304 and 371 °C) are displaced to different temperature values in the DTG curve of the mixture (323 and 342 °C) due to the impurity effect. The OLM melting is seen at 179 °C on the DSC curve of the mixture and the KP decomposition at 234 °C (Figure 8e and Table 1). The value of the overall experimental mass loss corresponds with the theoretical one, and these data support the hypothesis that no interaction occurred in the mixture.

The thermoanalytical profile of MgSTR exhibits a complex decomposition process, including two dehydration stages with a mass loss of about 3%. Above the 250 °C temperature value, multiple signals are observed on the DTG curve corresponding to the thermal degradation of the excipient that leads to a residual mass of 40.02% at 400 °C. The TG and DTG thermal curves of the KP + MgSTR mixture show the dehydration processes of the excipient with DTG_peaks_ at 81 and 98 °C. At temperature values higher than 153 °C, the thermal decomposition of KP and the excipient overlaps and a shift in DTG_peaks_ to lower temperature values is noticed in the DTG curve of the mixture. Also, a difference between the experimental and theoretic total mass loss values is observed, with the mass residue at 400 °C being 40.61%, a slightly higher value than the calculated one (33.64%). Moreover, two new endothermic events with maxima at 128 and 195 °C are noticed on the DSC curve of the mixture alongside those corresponding to the IC (DSC_peak_ at 172 and 224 °C) and excipient (DSC_peaks_ at 87 and 112 °C), all displaced to lower temperature values due to the impurity effect; these new peaks on the DSC curve together with the difference between theoretical and experimental mass residues provide evidence for the formation of intermediary products presenting different thermal behavior than the pure compounds. These data suggest the compatibility between the IC and the excipient at low temperature values, but above 120 °C, the interaction can be noticed.

The TG/DTG thermal curves of LA show the decomposition of the excipient in three stages; the first one corresponds to the LA dehydration in the temperature range of 95–150 °C, followed by a continuous mass loss between 200 and 400 °C, with a residual mass of 24.37% at 400 °C. The thermal profile of the OLM/DMβCD KP + LA exhibits dehydration of the excipient with the displacement of DTG_peak_ to lower temperature value (from 145 °C in the pure LA DTG curve to 133 °C in the DTG profile of the mixture) owing to the impurity effect. The mass loss value corresponding to the excipient dehydration (1.24%, Table 1) is lower than the expected theoretical one (2.45%). Moreover, the decomposition of the mixture above 175 °C shows an important mass loss process up to 278 °C, with a Δ*m* value that exceeds the corresponding ones for the individual components and a DTG_peak_ at a higher temperature value (251 °C) as compared to those of the individual systems (234 °C for KP and 237 °C for LA). Also, the excipient DTG_peak_ from 306 °C presents an important reduction in its intensity in the DTG curve of KP + LA (shown at 299 °C), suggesting a Δ*m* value lower in the temperature range of 278–311 °C than the one expected. The DSC curve of the mixture reveals a significant reduction in the endothermic processes corresponding to LA dehydration and decomposition (DSC_peaks_ at 138 and 213 °C), and also the occurrence of a new endothermic peak at 235 °C without a correspondent signal in DSC curves of the pure compounds. These data suggest a different thermal behavior of OLM/DMβCD KP + LA from those of the individual components, highlighting a lower stability of the mixture at temperature values above 164 °C. All these indicate no interaction between the IC and LA only at temperature values below 100 °C.

#### 3.5.2. FTIR Analysis

FTIR spectroscopy is a valuable technique for the investigation of potential physicochemical interactions between the drug substances and pharmaceutical excipients under ambient temperature, providing important information related to the chemical moiety to avoid in excipients for the obtaining of safe, stable and efficient pharmaceutical formulations [58].

**Figure 9:**
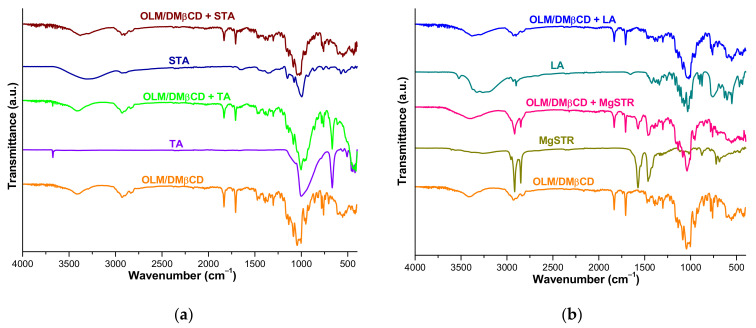
UATR-FTIR spectra of (**a**) OLM/DMβCD IC, TA, STA, and the physical mixture of IC with TA and STA; (**b**) OLM/DMβCD IC, MgSTR, LA, and the mixture of IC with MgSTR and LA, recorded at ambient temperature.

The FTIR spectra of OLM/DMβCD IC, selected excipients, and physical mixtures of KP with each excipient are depicted in Figure 9.

The FTIR spectrum of OLM/DMβCD IC reveals a broad band in the spectral region of 3530–3300 cm^−1^ (maximum at 3422 cm^−1^) that corresponds to the O-H stretching vibration from hydroxyl groups and other characteristic bands noticed in Table 2, which were mentioned in Section 3.4.2.

The FTIR spectral pattern of OLM/DMβCD + TA displays all the characteristic bands of KP, most of which are at the same wavenumbers as in the KP spectrum; the KP bands from 3422, 1044, 950, and 706 cm^−1^ appeared slightly shifted (at 3421, 1043, 952, and 704 cm^−1^) in the mixture spectrum. Also, TA presents a sharp characteristic band at 667 cm^−1^ (Figure 9a and Table 2) that overlaps with the KP bands from 679 and 660 cm^−1^, making them difficult to visualize in the mixture spectrum. No new bands are seen in the spectrum, with these data demonstrating the lack of interaction between KP and TA.

The absorption bands identified in the spectral pattern of the mixture between KP and STA (Table 2) belong in their majority to OLM/DMβCD; several are slightly displaced to different wavenumbers, but no disappearance of KP absorption bands or new signals were noted in the mixture spectrum.

The FTIR spectrum of OLM/DMβCD + MgSTR displays, in the spectral region of 3020–2860 cm^−1^, the sharp excipient bands from 2916 and 2849 cm^−1^ that mask the KP signals from 2928 and 2835 cm^−1^. All other spectral bands of the mixture correspond either to IC or the excipient (1570, 1375, and 721 cm^−1^), with the overall characteristics of the OLM/DMβCD + MgSTR spectrum supporting the hypothesis of a lack of interaction between the components.

The spectral data presented in Table 2 show the presence of the characteristic absorption bands of OLM/DMβCD in the spectrum of the OLM/DMβCD KP + LA mixture (Figure 9b) either at the same wavenumbers as in KP or slightly displaced to different wavenumbers, as well as the broad band of the excipient in the spectral range of 3550–3040 cm^−1^ that overlaps with the KP signal from 3422 cm^−1^.

The data obtained from FTIR studies reveal that no new signals are seen, and also, no disappearance of KP bands was noticed in the spectrum of the investigated binary mixtures, indicating a lack of interactions between components of the mixtures at ambient temperature. This leads to the conclusion that potential interactions shown by thermal investigations in the cases of OLM/DMβCD KP + MgSTR and OLM/DMβCD KP + LA are thermally induced.

#### 3.5.3. PXRD Studies

In this study, PXRD was used as a complementary technique for assessing the potential interactions between the IC and excipients.

**Figure 10:**
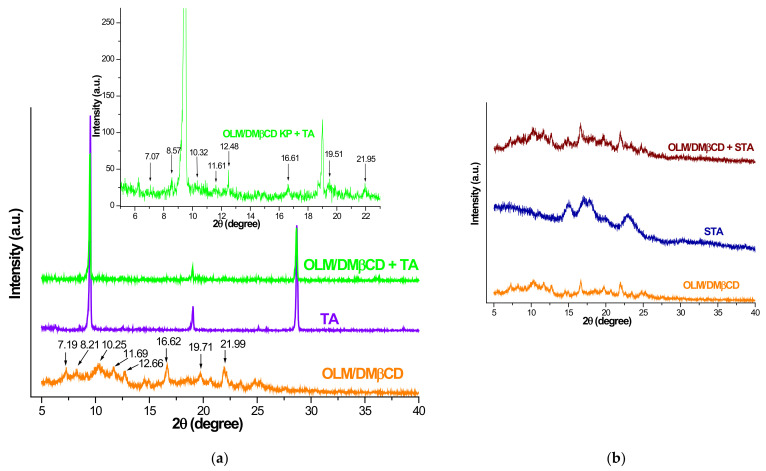

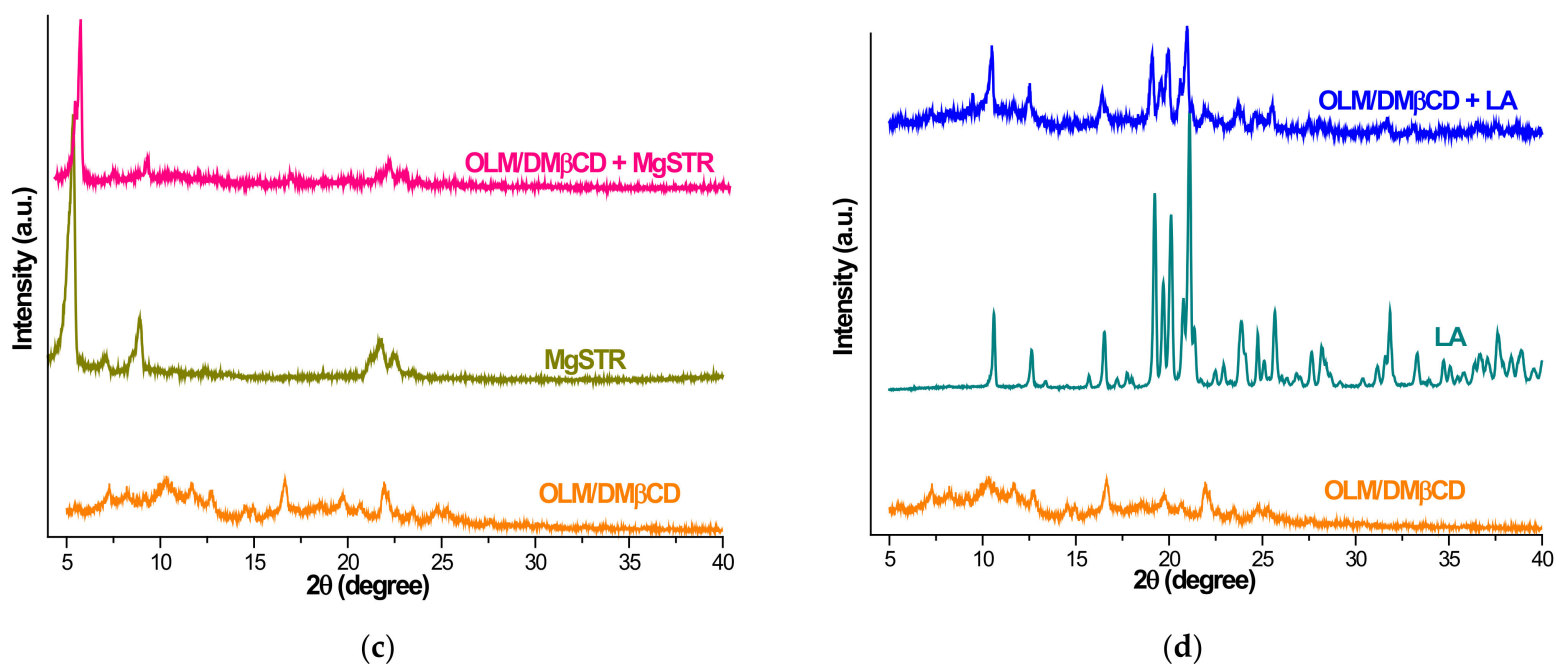
PXRD diffraction patterns of (**a**) OLM/DMβCD IC, TA, and their corresponding physical mixtures—main image; OLM/DMβCD KP + TA with 2θ values of diffraction peaks corresponding to KP—inset image; and OLM/DMβCD KP, excipients, and their mixture. (**b**) STA. (**c**) MgSTR. (**d**) LA.

The X-ray diffraction profiles of OLM/DMβCD IC, selected excipients, and their physical mixtures are depicted in Figure 10a–d.

The diffractogram of OLM/DMβCD KP exhibits two main characteristic reflections at 16.62 and 21.99 2*θ* and several peaks of low intensity at 2*θ* angles of 7.19, 8.21, 10.25, 11.69, 12.66, 16.62, 19.71, and 21.99. The diffraction pattern of the mixture of IC with TA reveals the presence of TA crystalline reflection from 9.47, 12.48, 19.01, and 28.65 2*θ*, as well as the peaks of the IC at 7.07, 8.57, 10.32, 11.61, 16.61, 19.51, and 21.96 2*θ*, some slightly shifted to different 2*θ* values (Figure 9a), suggesting no interaction between IC and the excipient. The diffraction profile of OLM/DMβCD + STA (Figure 9b) presents all the characteristic peaks of IC at 7.19, 8.21, 10.34, 11.62, 12.71, 16.61, 19.69, and 21.92 2*θ*, which overlap over the broad bands of STA that indicate its amorphous nature, highlighting a lack of interactions between the KP and STA.

In the diffraction profile of OLM/DMβCD + MgSTR, the characteristic reflections of the excipient are the most prominent, being noticed at 5.38, 7.17, 8.93, 21.82, and 22.55 2*θ*, slightly shifted from the 2*θ* values of pure MgSTR (5.35, 7.14, 8.95, 21.72, and 22.48). Although the intensity of IC peaks was significantly lower compared to that of excipient reflections, two IC characteristic peaks are noticed at 2*θ* values of 11.62 and 12.64 in the mixture diffractogram, while the rest of the IC peaks overlap over prominent excipient characteristic reflections, confirming the lack of interaction between the components of the mixture under ambient conditions. These results are in agreement with the outcomes of FTIR spectroscopy analysis and indicate the thermally induced interaction between IC and MgSTR.

The PXRD pattern of OLM/DMβCD + LA reveals the characteristic crystalline reflections of LA at 2*θ* values of 10.49, 12.53, 16.42, 19.09, 19.58, 19.93, 20.60, 20.96, 23.63, 24.57, 25.53, 31.70, and 33.11, slightly shifted as compared with those of pure LA (at 10.59, 12.62, 16.54, 19.22, 19.69, 20.11, 20.77, 21.09, 23.87, 24.75, 25.61, 31.85, and 33.32 2*θ*). Only two peaks of IC are visible in the diffractogram of the mixture, namely at 11.55 and 21.86 2*θ*, and the excipient characteristic reflections that are much more intense than those of IC are superimposed on the other IC peaks. No new peaks were observed, indicating no interaction between KP and LA under ambient conditions.

## 4. Conclusions

In this study, the inclusion complexation of OLM with DMβCD was carried out for improving the drug solubility, and also, the compatibility of the obtained IC with several pharmaceutical excipients was investigated. The kneading method was employed for the preparation of OLM/DMβCD IC, and various techniques confirmed its successful formation. The IC stoichiometry was 1:1 as the phase solubility analysis indicated. An important amorphization process of OLM in the IC was noticed, and the OLM solubility increased by 1.83-fold as compared to the pure drug due to the OLM encapsulation by DMβCD, emphasizing the solubilizing role of CD.

The thermoanalytical methods highlighted the compatibility of OLM/DMβCD KP with TA and STA and suggested interactions between IC and two excipients, namely MgSTR and LA, at temperature values above 120 and 100 °C, respectively. FTIR spectroscopy and PXRD results indicated a lack of interaction between IC and all tested excipients at ambient temperature, pointing out a thermally induced incompatibility of IC with MgSTR and LA. These results suggest that precautions should be considered regarding the utilization of MgSTR and LA as excipients in the development of new pharmaceutical dosage forms of OLM formulated as IC if, during the manufacturing process, temperatures exceed 100 °C.

## Figures and Tables

**Table 1:** The thermoanalytical data for OLM/DMβCD IC and its mixtures with excipients in synthetic air atmosphere and a heating rate of 10 °C min^−1^.

Sample	TG		Δm (%)	Mass Residue (%)	DTG		DSC	
	T_onset_ (°C)	T_offset_ (°C)			T_onset_ (°C)	T_peak_ (°C)	T_onset_ (°C)	T_peak_ (°C)
OLM/DMβCD KP	120	155	+0.10	27.25	-	-	155	180; 234
	165	291	9.70		165	183; 234		
	291	400	62.30		291	371		
OLM/DMβCD KP + TA	170	273	4.61	61.87	170	231	154	180; 234
	273	400	33.52		268	339		
OLM/DMβCD KP + STA	30	128	4.98	25.28	30	44	35	71
	175	255	3.38		175	231	155	179; 234
	255	400	66.37		255	323; 342		
OLM/DMβCD KP + MgSTR	70	89	0.41	40.61	70	81	71	87
	89	112	0.91		89	98	96; 122	112; 128
	153	240	5.27		112	216	153; 183	172; 195; 224
	240	400	52.80		240	327		
OLM/DMβCD KP + LA	106	150	1.24	26.42	106	133	93	137
	164	278	26.06		164	251	167; 201	179; 213; 235
	278	311	8.96		278	299		
	311	400	37.32		311	353		

**Table 2:** FTIR characteristic bands for OLM/DMβCD IC and its mixtures with selected excipients.

Sample	FTIR Spectral Regions (cm^−1^)		
	4000–2700	2000–1000	1000–650
OLM/DMβCD KP	3422; 2928; 2835	1832; 1707;1474; 1400; 1389; 1364; 1329; 1302; 1225; 1196; 1157; 1136; 1086; 1044; 1003	966; 950; 924; 856; 826; 783; 768; 760; 741; 706; 679; 660
OLM/DMβCD KP + TA	3676; 3421; 2928; 2835	1832; 1707;1474; 1400; 1389; 1364; 1329; 1302; 1227; 1196; 1157; 1136; 1086; 1043; 1003	966; 952; 924; 858; 826; 783; 768; 760; 741; 704; 667
OLM/DMβCD KP + STA	3422; 3377; 2932; 2832	1832; 1707; 1474; 1400; 1389; 1362; 1331; 1302; 1225; 1196; 1157; 1136; 1086; 1043; 1003	966; 953; 924; 899; 858; 826; 781; 768; 760; 741; 706; 679; 660
OLM/DMβCD KP + MgSTR	3418; 2916; 2849	1832; 1707; 1570; 1464; 1402; 1389; 1375; 1364; 1321; 1302; 1227; 1194; 1157; 1136; 1086; 1040; 1003	966; 953; 924; 858; 826; 783; 768; 760; 741; 721; 704; 681; 658
OLM/DMβCD KP + LA	3381; 3292; 2930; 2900; 2835	1832; 1707;1474; 1400; 1389; 1362; 1331; 1302; 1225; 1198; 1157; 1136; 1115; 1084; 1038; 1018; 1003	966; 953; 924; 899; 878; 858; 826; 781; 768; 760; 741; 706; 679; 658

## Data Availability

Data are contained in the manuscript.
